# Compound Xuanju capsules combined with phosphodiesterase-5 inhibitors as a new strategy for erectile dysfunction: a meta-analysis and trial sequential analysis

**DOI:** 10.3389/fphar.2025.1537789

**Published:** 2025-06-26

**Authors:** Ronghui Li, Min Liao, Yunfeng Yu, Bing Guo, Rong Yu, Qinghu He, Guomin Zhang

**Affiliations:** ^1^ School of Traditional Chinese Medicine, Hunan University of Chinese Medicine, Changsha, Hunan, China; ^2^ Department of Anesthesiology, People’s Hospital of Ningxiang City, Changsha, Hunan, China; ^3^ School of Traditional Chinese Medicine, Hunan University of Medicine, Huaihua, Hunan, China; ^4^ School of Integrated Chinese and Western Medicine, Hunan University of Chinese Medicine, Changsha, Hunan, China

**Keywords:** compound Xuanju capsule, phosphodiesterase type 5 inhibitor, erectile dysfunction, meta-analysis, trial sequential analysis

## Abstract

**Objective:**

The efficacy of compound Xuanju capsule (CXC) in the treatment of erectile dysfunction (ED) remains unclear. This study aimed to quantitatively assess the benefits and risks of CXC in the treatment of ED.

**Methods:**

Eight major databases were systematically searched for relevant literature published till 10 May 2025. Studies were screened based on established criteria; meta-analysis and trial sequential analysis of included literature were conducted.

**Results:**

The meta-analysis demonstrated that, compared with phosphodiesterase type 5 inhibitors alone, the combination of CXC and phosphodiesterase type 5 inhibitors significantly improved the International Index of Erectile Function (IIEF)-5 score by 3.19 points (mean difference [MD] = 3.19; 95% confidence interval [CI]: 2.42–3.96; *P* < 0.00001), clinical effectiveness rate by 23% (risk ratio [RR] = 1.23; 95% CI: 1.15–1.32; *P <* 0.00001), penile cavernous blood flow by 5.21 cm/s (MD = 5.21; 95% CI: 4.43–6.00; *P <* 0.00001), and serum testosterone levels by 4.09 nmol/L (MD = 4.09; 95% CI: 3.14–5.04; *P <* 0.00001). There was no significant difference in total adverse events between the groups (RR = 0.94; 95% CI: 0.62–1.42; *P* = 0.77). Trial sequential analysis confirmed that the meta-analysis results for IIEF-5, clinical effectiveness rate, penile cavernous blood flow, and serum testosterone levels were conclusive. However, the results of adverse events require further validation through additional similar studies. Funnel plot analysis and Egger’s test indicated no potential publication bias for outcomes other than the clinical effectiveness rate.

**Conclusion:**

CXC improves erectile function and testosterone levels in patients with ED without increasing the incidence of adverse events. These findings support the potential role of CXC as an adjunctive treatment for ED. However, due to the limitations in the quality of the current evidence, further validation through multicenter, randomized, double-blind controlled trials is necessary.

## 1 Introduction

Erectile dysfunction (ED) is a common disorder in men, characterized by the inability to achieve or maintain a sufficient erection for satisfactory sexual performance (Hatzimouratidis et al., 2010). The etiology of ED is multifactorial, including vasogenic, neurogenic, endocrine, pharmacologic depression, systemic diseases, and local penile injury (Lizza and Rosen, 1999). ED not only adversely affects male psychological wellbeing and mutual sexual satisfaction but also poses a potential threat to family harmony (Amoo et al., 2017). An epidemiological survey in 2,000 estimated that approximately 150 million people suffer from ED worldwide, a figure projected to rise to 322 million by 2025 (McKinlay, 2000; Shamloul and Ghanem, 2013). The combined prevalence of ED in adult males has been reported to exceed 20%, trending toward younger age (Nguyen et al., 2017), with ED patients aged 20–40 years accounting for 30% of the total number of patients (Huang et al., 2023). Phosphodiesterase-5 inhibitors (PDE5Is) are the first-line pharmacological treatment for ED, primarily by enhancing the bioavailability of nitric oxide (NO) (Mehrotra et al., 2007). However, PDE5Is do not cure ED and merely provide symptomatic relief. Additionally, while improving blood supply to the penis, PDE5Is also affect the function of tissues, such as blood vessels, smooth muscle in the airway, and skeletal muscle, which increases the risk of potential adverse events (Carson and Lue, 2005). PDE5Is may also increase the psychological burden of patients, and psychological resistance to PDE5Is has been reported in several patients with ED (Cai et al., 2020). Therefore, there is a need to develop a safe, long-term, and effective adjunctive treatment strategy for ED.

Compound Xuanju capsule (CXC) a traditional Chinese medicine preparation for treating ED, consisting of Black Ant [Formicidae; *Camponotus* spp], Epimedii folium [Berberidaceae; *Epimedium brevicornu Maxim*], Lycii Fructus [Solanaceae; *Lycium barbarum* L.], and Cnidii Fructus [Apiaceae; *Cnidium monnieri* (L.) Cuss] (Wang et al., 2020). Previous animal studies have shown that CXC improves erectile function by increasing serum testosterone and luteinizing hormone levels, as well as enhancing the index of accessory reproductive organs (Zhou et al., 2011). Additionally, pharmacological research indicates that CXC suppresses inflammatory responses by downregulating levels of TNF-α, IL-1β, and IL-17, thereby alleviating damage caused by prostatitis and autoimmune arthritis (Li et al., 2014; Wang et al., 2015; Wang CY. et al., 2016). Quantitative analysis using the multi-metabolites by single marker (QAMS) method has identified the main active metabolites of CXC as icariin, baohuoside I, osthole, catechin, epicatechin, bergapten, and imperatorin (Wang et al., 2020). Among these, icariin has been identified as the main active metabolite of CXC, with a content of over 4.0 mg/g (Ling, 2014). As research has progressed, the role of CXC in male diseases, especially ED, has received increasing attention. Previous studies have shown that CXC increases erectile hardness and duration in patients with ED, thereby improving the quality of their sexual lives (Wang et al., 2022). Additionally, CXC has been used to treat ED in conjunction with other male conditions, such as prostatitis and premature ejaculation (Liu et al., 2019; Hu et al., 2023). However, owing to the lack of high-quality evidence-based data, the specific benefits and risks of combining CXC with PDE5Is for the treatment of ED are unclear. In the present study, we performed a meta-analysis and trial sequential analysis (TSA) to evaluate the efficacy and safety of CXC, and to provide an evidence-based rationale for its use in the treatment of ED.

## 2 Phytochemical information of CXC

The CXC investigated in this study is a traditional Chinese medicine preparation produced by Cnstrong Company, with the national medicine approval number Z20060462. Currently, all CXC available on the market are original research drugs, and no generic versions have been approved for sale. CXC is composed of Black Ant [Formicidae; *Camponotus* spp], Epimedii folium [Berberidaceae; *Epimedium brevicornu Maxim*], Lycii Fructus [Solanaceae; *Lycium barbarum* L.], and Cnidii Fructus [Apiaceae; *Cnidium monnieri* (L.) Cuss]. The QAMS method has identified icariin, baohuoside I, osthole, catechin, epicatechin, bergapten, and imperatorin as the main active metabolites of CXC (Wang et al., 2020). Icariin is identified as the main active metabolite of CXC, with a content of over 4.0 mg/g (Ling, 2014). Detailed information on the CXC formulations used in each study is provided in Table 1.

**TABLE 1:** Metabolites and basic information of CXC.

Composition			Main active metabolites	Standard
Drug	Scientific name	Family		
Black ant	*Camponotus* spp.	Formicidae	Icariin, baohuoside I, osthole, catechin, epicatechin, bergapten, and imperatorin	Icariin >4.0 mg/g
Epimedii folium	*Epimedium brevicornu Maxim*.	Berberidaceae		
Lycii fructus	*Lycium barbarum* L.	Solanaceae		
Cnidii fructus	*Cnidium monnieri* (L.)Cuss.	Apiaceae		

CXC, compound Xuanju capsules.

Icariin can reduce the damage to endothelial and smooth muscle cells in the corpus cavernosum by inhibiting pyroptosis and apoptosis in rat corpus cavernosum tissue, thereby improving erectile function in diabetic rats (Yang et al., 2025). Additionally, icariin and baohuoside can relax corpus cavernosum smooth muscle by activating the NO/phosphodiesterase type 5/cyclic guanosine monophosphate (NO/PDE5/cGMP) signaling pathway, thus exerting a therapeutic effect on ED (Li et al., 2022). Osthole has the ability to upregulate the expression of hydrogen sulfide (H_2_S) generating enzymes and promote the endogenous synthesis of H_2_S, which is considered a key gaseous signaling molecule that facilitates smooth muscle relaxation (Alan-Albayrak et al., 2025). This suggests that osthole can regulate smooth muscle relaxation by promoting the synthesis and release of H_2_S, thereby improving erectile function (Alan-Albayrak et al., 2025). Catechin and epicatechin are both flavonoids that exhibit binding affinities for PDE5, comparable to that of sildenafil (Ejeje et al., 2024). Catechin and epicatechin can treat ED by inhibiting oxidative stress damage in endothelial and smooth muscle cells of the corpus cavernosum (Wang et al., 2024; Smith et al., 2023). Although there are no direct reports on the use of bergapten and imperatorin in the treatment of ED, both metabolites have demonstrated anti-inflammatory and antioxidant properties (Xu et al., 2025; Feng et al., 2025). In summary, as main active metabolites of CXC, icariin, baohuoside, osthole, catechin, and epicatechin can improve erectile function, while bergapten and imperatorin may have a positive impact on ED through their anti-inflammatory or anti-oxidative properties.

## 3 Materials and methods

This study strictly followed the Preferred Reporting Items for Systematic reviews and Meta-Analyses (PRISMA) guidelines (Page et al., 2021) and has been registered in PROSPERO (CRD420251072306, www.crd.york.ac.uk/PROSPERO/view/CRD420251072306).

### 3.1 Literature search

The search strategy for this study was developed using a combination of subject terms and free words, with the search field limited to the title or abstract. The search formula used was: ([Xuanju] AND [Erectile Dysfunction OR Male Impotence OR Male Sexual Impotence OR Impotence]). Literature related to the treatment of ED with CXC was retrieved from four public Chinese databases (China National Knowledge Infrastructure, VIP, Wanfang, and Sinomed), and four public English databases (Embase, PubMed, Cochrane Library, and Web of Science), with no restrictions on language, region, or other factors. The initial search was conducted on 31 February 2024, followed by an updated search on 10 May 2025.

### 3.2 Inclusion and exclusion criteria

The inclusion criteria were as follows: (i) the study design was a randomized controlled trial (RCT); (ii) patients had a diagnosis of ED (Zhang et al., 2016); (iii) the control group received PDE5Is, including sildenafil, vardenafil, tadalafil, lodenafil, avanafil, udenafil, mirodenafil, and other similar drugs; (iv) the experimental group received treatment with CXC in combination with PDE5Is, and the PDE5Is used were the same as those in the control group; (v) the efficacy endpoints included international index of erectile function-5 (IIEF-5) score, the clinical effectiveness rate, penile cavernous blood flow, and serum testosterone level, while the safety endpoint was the incidence of adverse events. The IIEF-5 is a widely utilized tool for assessing ED. It comprises five key questions that evaluate various aspects of erectile function, including confidence, hardness, and the ability to maintain an erection. The IIEF-5 is appreciated for its simplicity, cost-effectiveness, and robust scientific foundation, making it an essential instrument in clinical practice for both screening and managing ED. The clinical effectiveness rate is defined as the proportion of patients who report satisfaction with their erectile function following treatment. Additionally, penile cavernous blood flow and serum testosterone levels are important objective measures in the evaluation of erectile dysfunction. Penile cavernous blood flow, assessed through penile color Doppler ultrasound, is one of the key parameters for evaluating ED. Serum testosterone levels are positively correlated with libido and erectile function, making them a common indicator for investigating the causes of ED. Adverse events refer to any discomfort experienced by patients during the treatment period, including headaches, dizziness, nasal congestion, facial flushing, nausea, abdominal bloating, dyspepsia, gastrointestinal bleeding, liver function abnormalities, rashes, and other related symptoms.

The exclusion criteria were as follows: (i) research data published in duplicate; (ii) research data that were unusable; and (iii) non-comparable baseline information for the test and control groups.

### 3.3 Literature screening

The initial step involved importing the entire set of retrieved studies into a dedicated literature management software to facilitate efficient organization and screening. Predefined inclusion and exclusion criteria were applied to identify relevant studies. Two reviewers (RL and YY) independently performed the screening process based on titles and abstracts. Discrepancies in study selection were discussed collaboratively, and unresolved disagreements were resolved through consensus with a third reviewer (GZ). This rigorous screening process ensured the quality and relevance of the studies selected for further analysis.

### 3.4 Data collection

Following the screening process, the full texts of the eligible studies were retrieved for detailed data extraction. Two independent reviewers (RL and YY) systematically extracted key information from each study, including study characteristics, participant details, intervention details, measured outcomes, and main results. Any ambiguities or missing data encountered during this process prompted attempts to contact the original study authors for clarification. Consistency checks were performed to ensure data accuracy, and discrepancies between the two reviewers were discussed and resolved with the input of GZ.

### 3.5 Risk of bias assessment

The methodological quality of included studies was appraised using the Risk of Bias Tool provided by RevMan 5.3. The evaluation covered key domains such as sequence generation, allocation concealment, blinding of participants and personnel, blinding of outcome assessment, incomplete outcome data, selective reporting, and other potential biases. Each study was independently evaluated by RL and YY to minimize subjective bias. The assessments were rated as ‘low risk,’ ‘high risk,’ or ‘unclear risk’ for each domain. Any disagreements between the reviewers were addressed through discussion until consensus was reached, or GZ was consulted, if necessary. The results of this assessment informed the overall interpretation of study quality and the robustness of the meta-analytical findings.

### 3.6 Statistical analysis

The primary meta-analysis was conducted using RevMan 5.3. For continuous variables, the mean difference (MD) with 95% confidence interval (CI) was employed as the effect measure, whereas for dichotomous variables, the risk ratio (RR) with 95% CI was utilized. To assess heterogeneity across studies, the I^2^ statistic was calculated; an I^2^ value of <50% was considered indicative of low heterogeneity, while values ≥ 50% suggested substantial heterogeneity. Based on heterogeneity levels, either a fixed-effects model (for I^2^ < 50%) or a random-effects model (for I^2^ ≥ 50%) was selected to combine the data. The threshold for statistical significance was set at a two-tailed *P*-value of less than 0.05.

To further investigate the influence of clinical factors on the primary efficacy endpoints, predefined subgroup analyses were performed. These analyses focused on variables such as the dosage of CXC agents, the type of control medications used, and the duration of treatment. By stratifying the data accordingly, we aimed to identify potential variations in treatment effects attributable to these clinical factors, thereby enhancing the interpretability of our results. In addition, a leave-one-out sensitivity analysis was conducted to evaluate the robustness of the combined results. This method involved systematically removing one study at a time from the meta-analysis to assess how each individual study impacted the overall effect size. This process allowed us to determine whether any single study disproportionately influenced the results, thereby providing insights into the stability and reliability of our findings.

A sequential analysis of the data was performed using TSA software version 0.9 beta. This approach determines whether the accumulated evidence from the meta-analysis is conclusive. The results were considered definitive when the Z-value curve reached the boundary value, indicating that sufficient evidence had been accumulated to support the conclusions drawn from the meta-analysis.

Publication bias was assessed using funnel plots and Egger’s test. Funnel plots are graphical tools that plot the effect size of each study against a measure of its precision. Asymmetry in the funnel plot may indicate the presence of publication bias, where smaller studies with non-significant results are less likely to be published. Egger’s test, performed using Stata 15.0, is a statistical method to formally test for funnel plot asymmetry. The test generates a *P*-value, and a *P*-value > 0.1 was used as an indication that there was no significant publication bias. The non-significant P-value suggests that the observed asymmetry in the funnel plot could be due to chance rather than true publication bias.

Finally, we evaluated the certainty of the evidence using the Grading of Recommendations Assessment, Development and Evaluation (GRADE) approach, which considers factors such as study limitations, inconsistency, indirectness, imprecision, and publication bias. This comprehensive evaluation enabled a clear assessment of the overall quality of the evidence supporting our findings.

## 4 Results

### 4.1 Literature screening

**FIGURE 1:**
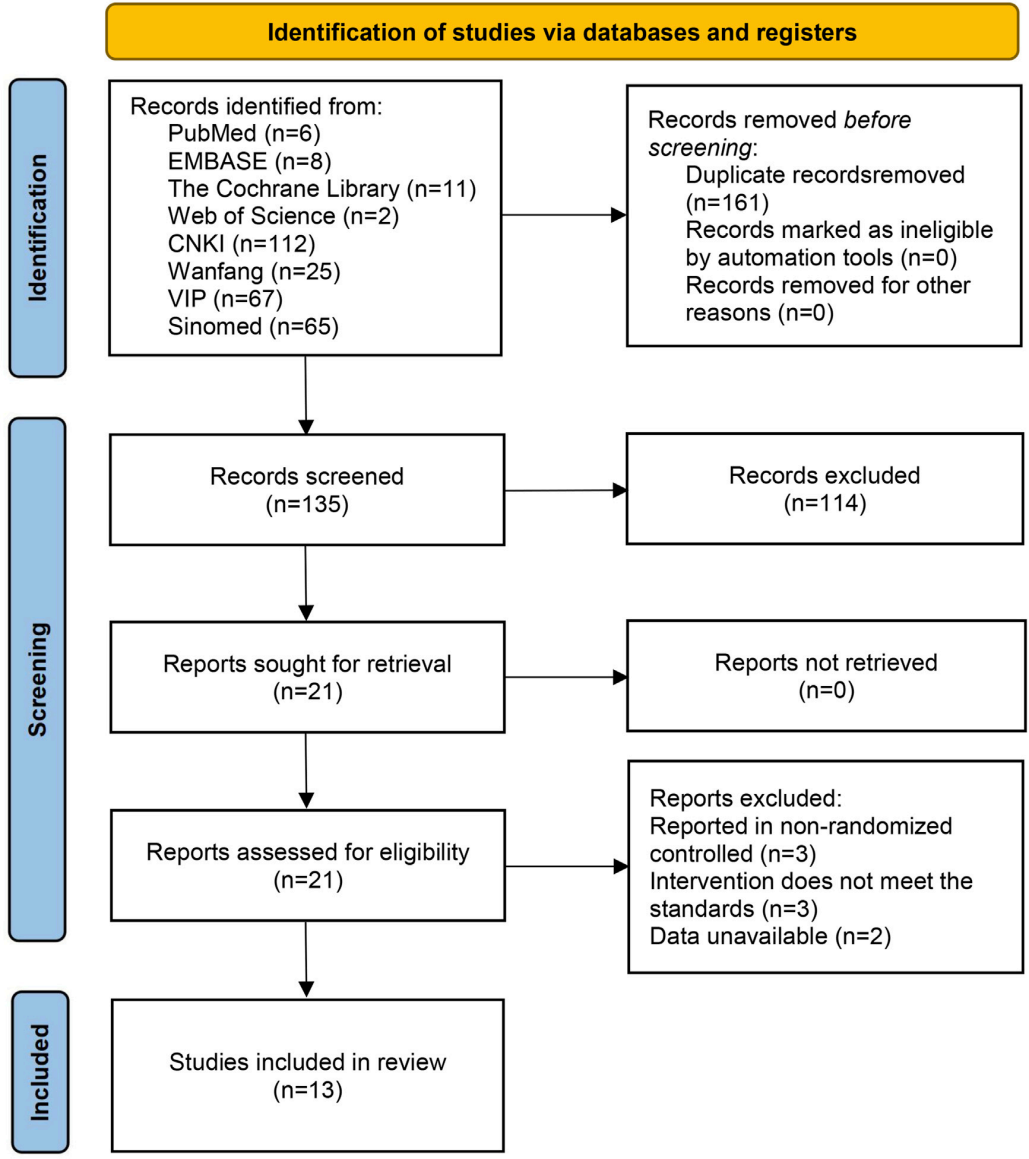
Literature screening process.

The database search initially identified 296 documents. During the screening process, 161 duplicate documents were excluded, and 122 documents were excluded based on the inclusion and exclusion criteria. Ultimately, 13 documents (Liu et al., 2019; Chen et al., 2012; Dai et al., 2018; Gao and Wang, 2016; Huang, 2020; Jiang et al., 2016; Liu, 2024; Nan et al., 2022; Sun, 2023; Wang et al., 2018; Wang, 2019; Wang Y. et al., 2016; Zhang, 2024) were included in the final analysis. The detailed process of literature screening is illustrated in Figure 1.

### 4.2 Basic characteristics

A total of 13 clinical studies (Liu et al., 2019; Chen et al., 2012; Dai et al., 2018; Gao and Wang, 2016; Huang, 2020; Jiang et al., 2016; Liu, 2024; Nan et al., 2022; Sun, 2023; Wang et al., 2018; Wang, 2019; Wang Y. et al., 2016; Zhang, 2024) were included in this review. All the experimental centers were located in China, the publication years were from 2012 to 2024, and the total sample size was 1,019 cases. Among them, 511 were treated with PDE5Is, while 508 were treated with CXC combined with PDE5Is. The characteristics of the included studies are shown in Table 2.

**TABLE 2:** Basic characteristics of the included studies.

Study	Sample (E/C)	Age(years)	Disease duration (months)	Intervention	Control	Treatment duration (weeks)
Chen et al. (2012)	18/18	40.2	—	CXC 0.84 g tid Sildenafil 50 mg	Sildenafil 100 mg	8
Dai et al. (2018)	42/45	42.9	—	CXC 1.26 g tid Tadalafil 10 mg	Tadalafil 10 mg	8
Gao and Wang (2016)	40/40	47.1	48.0	CXC 1.26 g tid Sildenafil 50 mg Vitamin E 10 mg tid	Sildenafil 50 mg Vitamin E 10 mg tid	4
Huang (2020)	40/40	41.8	—	CXC 1.26 g tid Tadalafil 10 mg	Tadalafil 10 mg	8
Jiang et al. (2016)	40/40	—	—	CXC 1.26 g tid Tadalafil 10 mg	Tadalafil 10 mg	4
Liu et al. (2019)	40/40	33.0	7.5	CXC 1.26 g tid Sildenafil 50 mg	Sildenafil 50 mg	6
Liu (2024)	62/62	40.2	8.7	CXC 1.26 g tid Tadalafil 5–20 mg	Tadalafil 5–20 mg	12
Nan et al. (2022)	43/43	31.5	7.4	CXC 1.26 g tid Tadalafil 10 mg	Tadalafil 10 mg	12
Sun (2023)	40/40	41.9	11.6	CXC 1.26 g tid Sildenafil 50 mg	Sildenafil 50 mg	12
Wang et al. (2018)	40/40	43.8	13.9	CXC 1.26 g tid Tadalafil 5 mg	Tadalafil 5 mg	4
Wang (2019)	31/31	39.8	—	CXC 1.26 g tid Tadalafil 10 mg	Tadalafil 10 mg	4
Wang Y. et al. (2016)	42/42	37.4	41.4	CXC 1.26 g tid Fatanafil 50 mg	Fatanafil 10 mg	8
Zhang (2024)	30/30	42.4	12.7	CXC 1.26 g tid Sildenafil 50 mg	Sildenafil 50 mg	4

Baseline information, including sex, age, and disease duration, was comparable between the experimental and control groups in each of the included studies. CXC, compound Xuanju capsules.

### 4.3 Risk of bias

**FIGURE 2:**
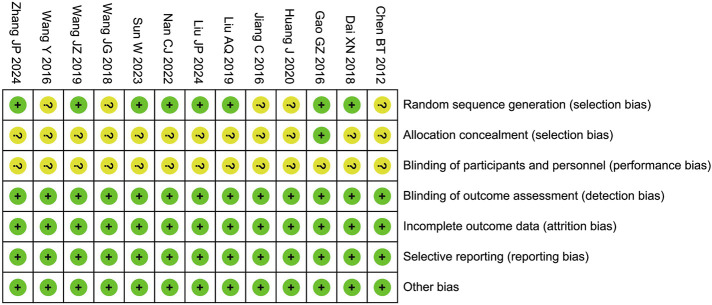
Risk assessment of bias.

In the risk of bias assessment, seven studies were flagged with an unclear risk level in the domain of random sequence generation. This uncertainty stemmed from the insufficient details regarding the randomization process provided in these studies. Regarding allocation concealment, 12 studies were deemed to have an unclear risk. The root cause was the lack of comprehensive information about the concealment methods employed, leaving the integrity of the allocation process open to question. In the domain of blinding of participants and personnel, 13 studies were assigned an unclear risk rating. The main issue was that these studies failed to mention the use of a placebo or the specific blinding techniques, which could potentially introduce bias into the research. On a more positive note, the included studies demonstrated a low risk of bias in several other critical areas. These areas encompass blinding of outcome assessment, incomplete outcome data, selective reporting, and other potential sources of bias, as shown in Figure 2.

### 4.4 Meta-analysis

#### 4.4.1 IIEF-5

**FIGURE 3:**
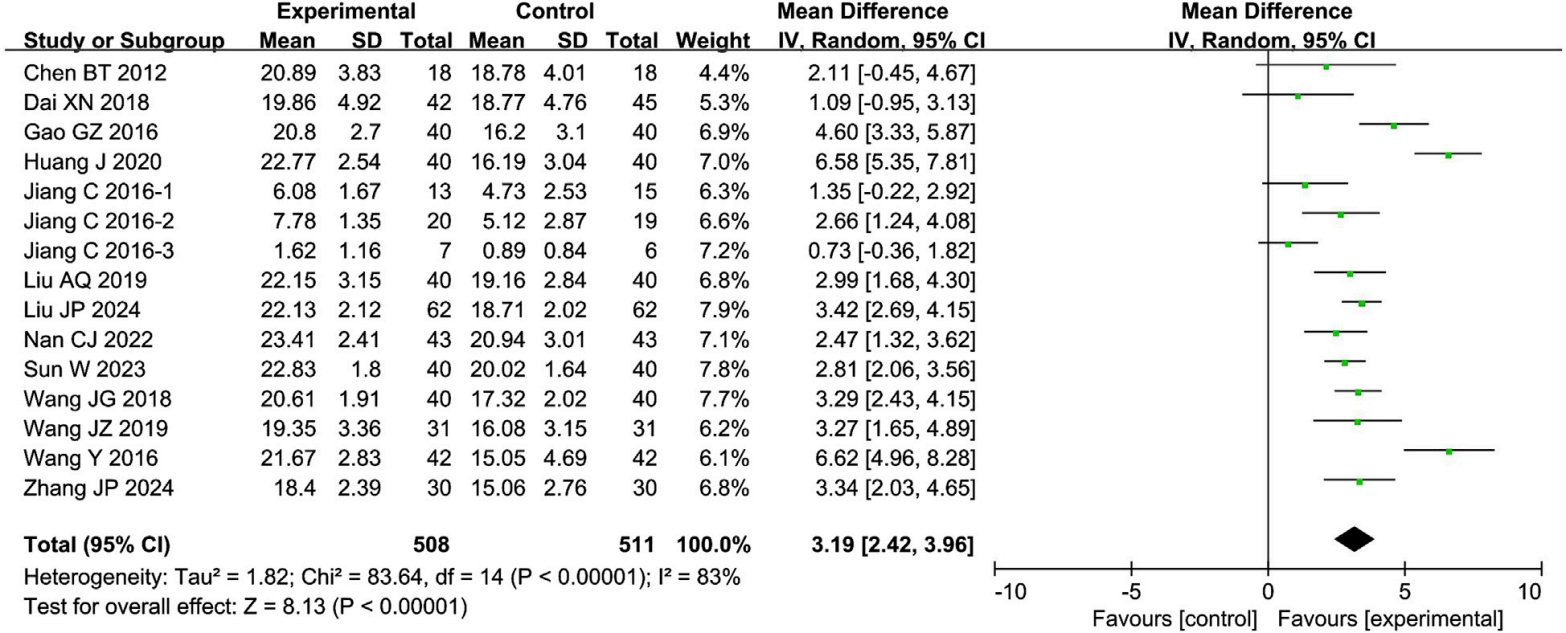
Forest plot of the meta-analysis on International Index of Erectile Function-5 (IIEF-5).

The meta-analysis of the IIEF-5 score included 13 studies with 1,019 participants. The results showed that the combination of CXC with PDE5Is significantly improved the IIEF-5 score by 3.19 points compared to PDE5Is alone (MD = 3.19; 95% CI: 2.42–3.96; *P <* 0.00001), as shown in Figure 3. The sensitivity analysis showed that the meta-analysis results of IIEF-5 were robust.

#### 4.4.2 Clinical effectiveness rate

**FIGURE 4:**
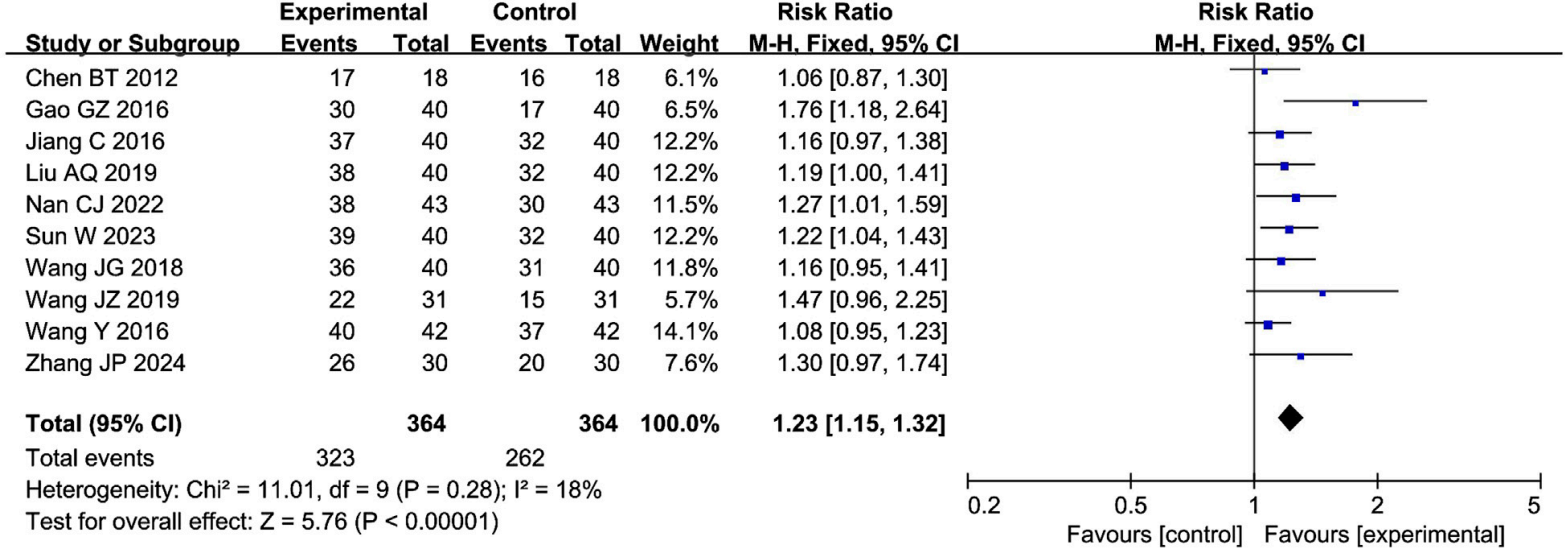
Forest plot of the meta-analysis on the clinical effectiveness rate.

The meta-analysis of the clinical effectiveness rate included 10 studies with 585 participants. The results showed that the combination of CXC with PDE5Is significantly increased the clinical effectiveness rate by 23% compared with PDE5Is (RR = 1.23; 95% CI: 1.15–1.32; *P <* 0.00001), as shown in Figure 4. The sensitivity analysis showed that the meta-analysis results of IIEF-5 were robust.

#### 4.4.3 Penile cavernous blood flow

**FIGURE 5:**
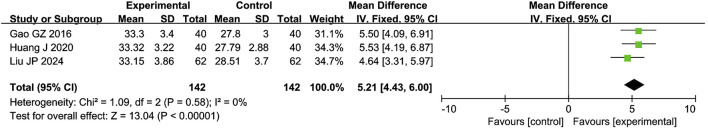
Forest plot of the meta-analysis on the penile cavernous blood flow.

The meta-analysis of penile cavernous blood flow included 3 studies with 284 participants. The results showed that the combination of CXC with PDE5Is significantly increased the penile cavernous blood flow by 5.21 cm/s compared with PDE5Is (MD = 5.21; 95% CI: 4.43–6.00; *P <* 0.00001), as shown in Figure 5. The sensitivity analysis showed that the meta-analysis results of penile cavernous blood flow were robust.

#### 4.4.4 Serum testosterone levels

**FIGURE 6:**
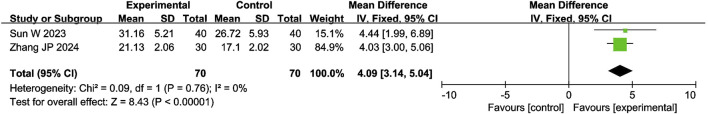
Forest plot of the meta-analysis on serum testosterone levels.

The meta-analysis of serum testosterone levels included 2 studies with 140 participants. The results showed that the combination of CXC with PDE5Is significantly increased the penile cavernous blood flow by 4.09 nmol/L compared with PDE5Is (MD = 4.09; 95% CI: 3.14–5.04; *P <* 0.00001), as shown in Figure 6. The sensitivity analysis showed that the meta-analysis results of serum testosterone levels were robust.

#### 4.4.5 Adverse events

**FIGURE 7:**
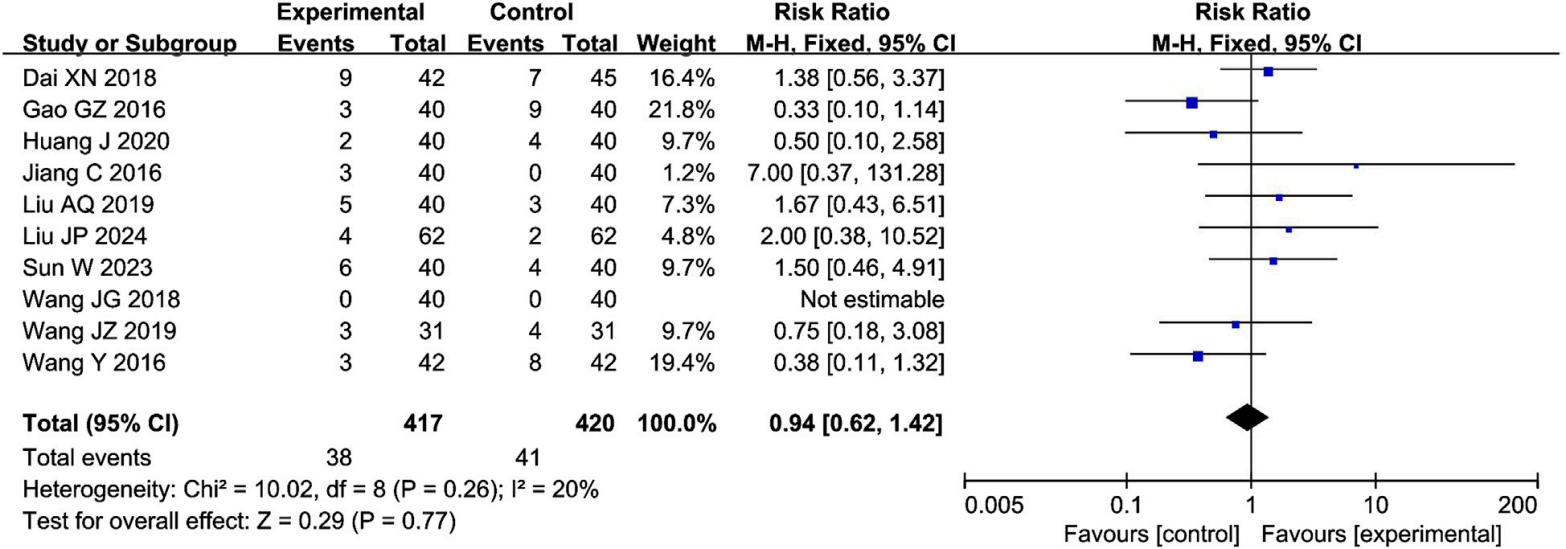
Forest plot of the meta-analysis on total adverse events.

The meta-analysis of adverse events included 10 studies with 837 participants. The results showed that the total adverse events were 9.11% (38/417) for the combination of compound CXC with PDE5Is and 9.76% (41/420) for the PDE5Is, which were comparable between the two groups (RR = 0.94; 95% CI: 0.62–1.42; *P* = 0.77), as shown in Figure 7. The sensitivity analysis showed that the meta-analysis results of adverse events were robust. In the analysis of individual adverse events, headache (RR = 0.73; 95% CI: 0.38–1.38; *P* = 0.33), dizziness (RR = 0.33; 95% CI: 0.01–7.96; *P* = 0.50), nasal congestion (RR = 0.39; 95% CI: 0.09–1.66; *P* = 0.20), facial flushing (RR = 1.00; 95% CI: 0.26–3.87; *P* = 1.00), nausea (RR = 1.80; 95% CI: 0.39–8.26; *P* = 0.45), abdominal distension (RR = 1.33; 95% CI: 0.30–5.87; *P* = 0.70), dyspepsia (RR = 1.33; 95% CI: 0.34–5.21; *P* = 0.68), gastrointestinal bleeding (RR = 0.33; 95% CI: 0.04–3.14; *P* = 0.34), liver dysfunction (RR = 0.50; 95% CI: 0.09–2.65; *P* = 0.42), and rash (RR = 5.00; 95% CI: 0.24–102.07; *P* = 0.30) were all comparable between the two groups, as shown in Table 3.

**TABLE 3:** Meta-analysis of individual adverse events associated with compound Xuanju capsules in the treatment of erectile dysfunction.

Adverse event	Experimental group	Control group	I^2^	RR (95% CI)	P value
Headache	14/337	20/340	0	0.73 (0.38, 1.38)	0.33
Dizziness	0/42	1/42	0	0.33 (0.01, 7.96)	0.50
Nasal congestion	2/124	6/127	0	0.39 (0.09, 1.66)	0.20
Facial flushing	4/82	4/82	0	1.00 (0.26, 3.87)	1.00
Nausea	4/111	2/111	0	1.80 (0.39, 8.26)	0.45
Abdominal distension	3/142	2/118	0	1.33 (0.30, 5.87)	0.70
Dyspepsia	4/115	3/118	0	1.33 (0.34, 5.21)	0.68
Gastrointestinal bleeding	0/80	2/80	0	0.33 (0.04, 3.14)	0.34
Liver dysfunction	2/80	4/80	0	0.50 (0.09, 2.65)	0.42
Rash	2/62	0/62	0	5.00 (0.24,102.07)	0.30

RR, risk ratio; CI, confidence interval.

### 4.5 Subgroup analysis

Subgroup analyses in this study were conducted to investigate the drug dosage, control drug, and treatment duration on the effects using IIEF-5 as an indicator. The results showed that in terms of dosage, 1.26 g/time of CXC significantly improved IIEF-5 score (MD = 3.24; 95% CI: 2.45–4.03; *P <* 0.00001), while 0.84 g/time CXC did not have a significant effect on the IIEF-5 score (MD = 2.11; 95% CI: -0.45–4.67; *P =* 0.11). In terms of control drugs, tadalafil (MD = 2.78; 95% CI: 1.79–3.76, *P <* 0.00001), sildenafil (MD = 3.26; 95% CI: 2.54–3.99; *P <* 0.00001), or vardenafil (MD = 6.62; 95% CI: 4.96–8.28; *P <* 0.00001) in combination with CXC significantly improved IIEF-5 scores. In terms of duration of treatment, both ≤6 weeks (MD = 2.78; 95% CI: 1.90–3.66; *P <* 0.00001) and >6 weeks (MD = 3.66; 95% CI: 2.35–4.98; *P <* 0.0001) of treatment with CXC significantly improved the IIEF-5 score, as shown in Table 4.

**TABLE 4:** Subgroup analysis of compound Xuanju capsules in the treatment of erectile dysfunction.

Subject	Subgroup	I^2^	MD (95% CI)	*P* value
CXC dosage	0.84 g/time	0	2.11 (−0.45, 4.67)	0.11
	1.26 g/time	84	3.24 (2.45, 4.03)	<0.00001
Control drug	Tadalafil	87	2.78 (1.79, 3.76)	<0.00001
	Sildenafil	38	3.26 (2.54, 3.99)	<0.00001
	Vardenafil	0	6.62 (4.96, 8.28)	<0.00001
Treatment duration	≤6 weeks	74	2.78 (1.90, 3.66)	<0.00001
	>6 weeks	88	3.66 (2.35, 4.98)	<0.00001

CXC, compound Xuanju capsules; CI, confidence interval; MD, mean difference.

### 4.6 TSA

**FIGURE 8:**
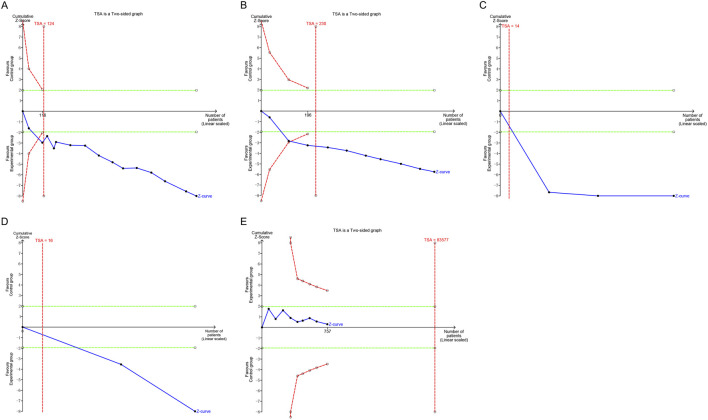
Trial sequential analysis of the efficacy and safety endpoints: **(A)** IIEF-5; **(B)** Clinical effectiveness rate; **(C)** Penile cavernous blood flow; **(D)** Serum testosterone levels; **(E)** Total adverse events. IIEF-5, International Index of Erectile Function-5.

In the present study, the TSA revealed that the Z-value curves for the IIEF-5 and the clinical effectiveness rate crossed the boundaries in the third and fourth studies, respectively. In contrast, both the penile cavernous blood flow and serum testosterone levels crossed the boundaries in the first study. These findings suggest that the meta-analysis results for these parameters are conclusive. Regarding adverse events, the cumulative Z-value was significantly smaller than the TSA boundary value of 83,577. This indicates that the results for adverse events require validation through more studies. Relevant details are presented in Figure 8.

### 4.7 Publication bias

**FIGURE 9:**
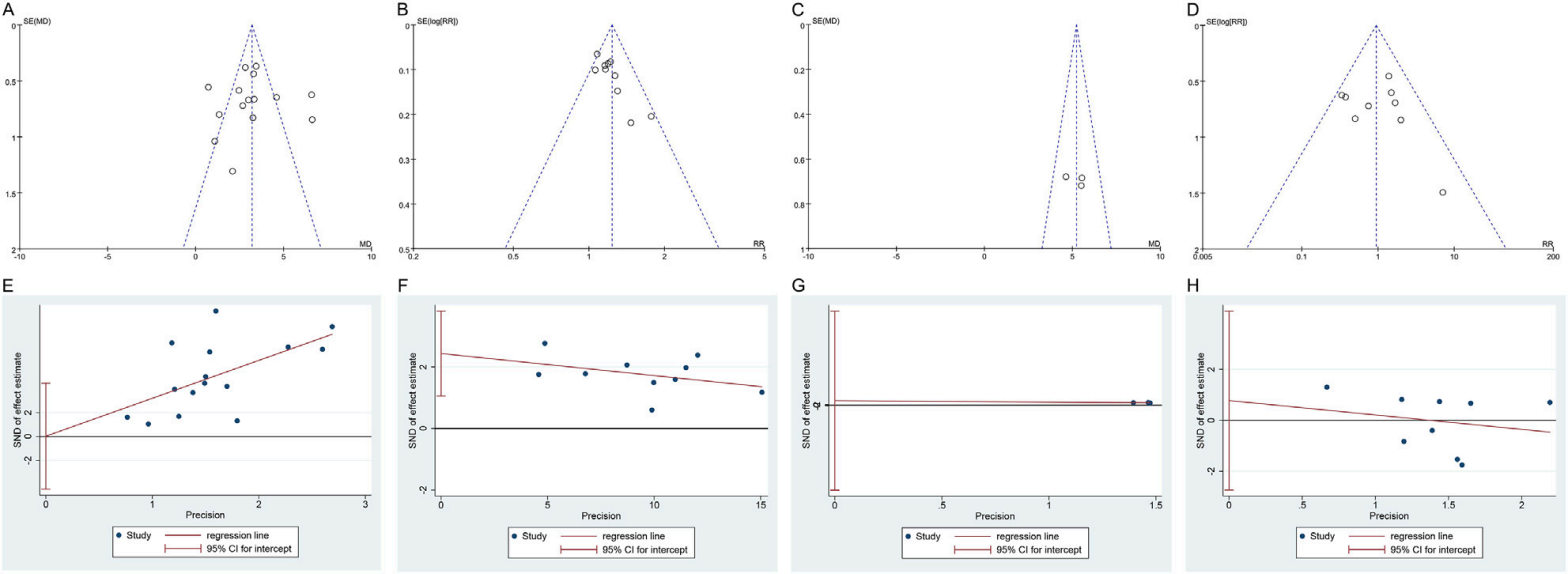
Funnel plot and Egger’s test of publication bias: **(A)** Funnel plot of IIEF-5; **(B)** Funnel plot of clinical effectiveness rate; **(C)** Funnel plot of penile cavernous blood flow; **(D)** Funnel plot of total adverse events; **(E)** Egger’s test of IIEF-5; **(F)** Egger’s test of clinical effectiveness rate; **(G)** Egger’s test of penile cavernous blood flow; **(H)** Egger’s test of total adverse events. IIEF-5, International Index of Erectile Function-5.

The funnel plots for IIEF-5, clinical effectiveness rate, and adverse events displayed an asymmetric scatter distribution, suggesting the presence of potential publication bias. In contrast, the funnel plot for penile cavernous blood flow showed a symmetrical scatter distribution, indicating no publication bias. The Egger’s test revealed no potential publication bias for IIEF-5 (*P =* 0.989), penile cavernous blood flow (*P =* 0.630), and adverse events (*P =* 0.617), suggesting that the observed publication bias in the funnel plots may not have a significant impact on the results. However, the Egger’s test also indicated the presence of potential publication bias for the clinical effectiveness rate (*P =* 0.004), as shown in Figure 9. Since only two studies were included for serum testosterone levels, the assessment of publication bias was not conducted.

### 4.8 Certainty of evidence

According to the GRADE system, the certainty of evidence for the IIEF-5, clinical effectiveness rate, penile cavernous blood flow, serum testosterone levels, and adverse events was determined to be low. Detailed results of this assessment are presented in Table 5.

**TABLE 5:** Certainty of evidence.

Outcome	Risk of bias	Inconsistency	Indirectness	Imprecision	Others	RR/MD (95% CI)	Certainty of evidence
IIEF-5	Serious	Serious	None	None	None	3.19 (2.42, 3.96)	Low
Clinical effectiveness rate	Serious	None	None	None	Publication bias	1.23 (1.15, 1.32)	Low
Penile cavernous blood flow	Serious	None	None	Serious	None	5.21 (4.43, 6.00)	Low
Serum testosterone levels	Serious	None	None	Serious	None	4.09 (3.14, 5.04)	Low
Adverse events	Serious	None	None	Serious	None	0.94 (0.62, 1.42)	Low

IIEF-5, International Index of Erectile Function-5; CI, confidence interval; MD, mean difference; RR, risk ratio.

## 5 Discussion

### 5.1 Research background and significance

ED is a common condition that leads to decreased quality of life and is an important cause of depression in men (Amoo et al., 2017). A European research study found that patients with ED had significantly lower physical and mental domain quality of life scores than men without ED (Jannini et al., 2014). A related meta-analysis showed a 192% increased risk of depression in men with ED, compared to those without ED (Liu et al., 2018). Given the potential detriment to mental health in men with ED, it is necessary to intervene aggressively. PDE5Is are widely recognized as first-line treatments for ED, as they maintain the cyclic guanosine monophosphate (cGMP) concentration in penile tissue by inhibiting PDE-5 activity, which in turn promotes NO bioavailability and facilitates the onset and maintenance of an erection (Omote, 1999). Although PDE5Is offer hope to patients with ED, their potential limitations and adverse events remain a concern. While PDE5Is provide temporary relief from the symptoms of ED, they cannot cure the disease itself (Cai et al., 2020). Long-term administration of PDE5Is not only leads to decreased sensitivity to the drug, but may also cause patients to develop resistance (Cai et al., 2020). Additionally, although most patients tolerate PDE5Is well, adverse events such as headache, dyspepsia, and visual abnormalities may still occur (Dhaliwal and Gupta, 2024). Therefore, it is important to explore adjunctive therapeutic strategies that can improve erectile function.

CXC is a traditional Chinese medicine preparation produced by Cnstrong company, and currently only the original drug with the national medicine approval number Z20060462 is circulating on the market. It is composed of Black Ant [Formicidae; *Camponotus* spp], Epimedii folium [Berberidaceae; *Epimedium brevicornu Maxim*], Lycii Fructus [Solanaceae; *Lycium barbarum* L.], and Cnidii Fructus [Apiaceae; *Cnidium monnieri* (L.) Cuss]. The main active metabolites include icariin, baohuoside I, osthole, catechin, epicatechin, bergapten, and imperatorin (Wang et al., 2020). In 2006, Cai et al. (Cai et al., 2006) published the first clinical trial of CXC for the treatment of ED, reporting promising therapeutic potential. There have subsequently been numerous positive reports on the use of CXC for the treatment of male disorders. Nevertheless, due to the lack of high-quality evidence-based data, the potential risks and relative benefits of CXC in patients with ED remain unclear. This study includes the first meta-analysis and TSA evaluating the combination of CXC with a PDE5I for treating ED, aiming to strengthen the clinical evidence supporting the use of CXC.

### 5.2 Evaluation of effectiveness

The IIEF-5 score is a widely used tool for diagnosing ED. Owing to its high sensitivity and specificity in the diagnosis of ED, the IIEF-5 score is widely used to both diagnose ED and determine its severity (Rc et al., 1997). Clinical effectiveness rate, defined as the percentage of people satisfied with erectile function relative to the total population, is important for assessing the effectiveness of a given treatment (Liu, 2016). The penile cavernous blood flow and serum testosterone levels are critical factors in the evaluation of ED, as they play pivotal roles in achieving and maintaining an erection (Hoppe et al., 2023; Moreno et al., 2011). Adequate penile blood flow is crucial for erectile function, as it directly responds to the physiological processes involved in achieving an erection (Hoppe et al., 2023). Additionally, low testosterone levels can adversely affect libido and contribute to ED by impairing the regulation of nitric oxide synthase (NOS) (Moreno et al., 2011). This meta-analysis showed that compared with PDE5I alone, CXC combined with PDE5Is significantly improved the IIEF-5 scores by 3.19, clinical effectiveness rate by 23%, penile cavernous blood flow by 5.21 cm/s, and serum testosterone levels by 4.09 noml/L, suggesting that the addition of CXC to PDE5I can effectively enhance and maintain erectile function.

Furthermore, CXC has also demonstrated the ability to reduce PDE5I dependence and maintain long-term effects. A study by Porter et al. (Chen et al., 2012) showed that the combination of CXC with 50 mg of sildenafil improved the IIEF-5 scores and Total Sexual Satisfaction (TSS) partner scores of patients more substantially over a 2-month treatment period than 100 mg of sildenafil alone. This suggests that a combination regimen containing CXC reduces the necessary PDE5I dosage and helps reduce patient dependence and psychological resistance to PDE5Is. Additionally, Nan et al. (Nan et al., 2022)found that 6 months after the discontinuation of treatment, patients with ED who had been treated with CXC for a period of 3 months still had better erectile function than the control group. These results suggest that CXC may have a long-term effect on improving erectile function, rather than a purely symptomatic treatment.

Additionally, the included studies reported that CXC also conferred benefits in terms of Erection Hardness Score (EHS), Sexual Encounter Profile Question 3 (SEP-Q3) score, Quality of Erection Questionnaire (QEQ), and TSS score. The EHS is a common tool used to assess erectile hardness; SEP-Q3 is an important measure of the ability of a patient to perform and maintain an erection during sexual intercourse; the QEQ is a questionnaire used to assess erectile function and quality of sexual life in men; and the TSS is an indicator of the satisfaction of patients and their sexual partners with erectile function and sexual life. The improvements in these indicators suggest that CXC effectively improves penile erection hardness, male sexual life quality, and partner sexual life satisfaction.

Subgroup analyses showed that in terms of dosage, 1.26 g/time of CXC significantly improved the IIEF-5 scores, while 0.84 g/time of CXC had no significant effect on IIEF-5 scores, suggesting that 1.26 g/time may be the lowest effective dose of CXC for the treatment of ED. In terms of control medications, combining CXC with tadalafil, sildenafil, or vardenafil improved the IIEF-5 scores of patients, suggesting that the benefit of CXC was not limited by the specific type of PDE5I. In terms of treatment duration, patients with ED treated with CXC for either less than or more than 6 weeks experienced improvements in IIEF-5 scores, suggesting that CXC may have both short- and long-term effects. This suggests that CXC can be flexibly paired with different PDE5I and is adaptable to different treatment durations.

### 5.3 Mechanism analysis

Penile erections involve the synergistic action of tissues, such as the vascular endothelium, autonomic nerves, bundles of smooth muscle cells, and fibroblasts. Following sexual stimulation, nerve impulses release neurotransmitters in the corpus cavernosum, leading to the production of NO by endothelial cells and its diffusion into the neighboring smooth muscle cells. Subsequently, NO stimulates vascular smooth muscle cells to synthesize cGMP, resulting in vasodilation and increased blood flow to the cavernous body of the penis, and, in turn, penile erection (McMahon, 2019). As a classical ED therapeutic drug, PDE5Is mainly reduce cGMP degradation by inhibiting PDE-5 activity, which, in turn, maintains penile blood supply and improves erectile function. The mechanism of action of CXC in treating ED may be related to the promotion of NO release and an increase in serum testosterone levels. CXC has been shown to increase serum NO and NOS levels. NOS plays a key role in NO synthesis, and generates NO by catalyzing the oxidation of L-arginine (Naseem, 2005). NO increases blood flow to the cavernous body of the penis by stimulating cGMP synthesis in vascular smooth muscle, thus maintaining the erectile state of the penis (Cooke and Losordo, 2002). Additionally, CXC has been reported to increase serum testosterone and follicle-stimulating hormone levels (Wu et al., 2013). Testosterone not only maintains the anatomical structure and physiological function of sex organs but also plays a role in central and peripheral penile erection regulation (Gerald and Raj, 2022). Therefore, this suggests that CXC may assist in regulating penile erectile function by increasing testosterone levels.

The primary active components of CXC include icariin, baohuoside I, osthole, catechin, epicatechin, bergapten, and imperatorin (Wang et al., 2020). Notably, icariin stands out as the principal active metabolite, with a concentration exceeding 4.0 mg/g (Ling, 2014). First, icariin enhances the transmembrane transport of cholesterol by upregulating the expression of StAR, P450C17, and the benzodiazepine receptor (PBR), which subsequently boosts testosterone synthesis (Chen et al., 2014). Additionally, it facilitates the neogenesis and stabilization of the vascular endothelium by increasing the expression of sphingosine-1-phosphate (S1P) and its receptor, S1PR1, thereby improving erectile function (Yao et al., 2023). Second, baohuoside I promotes relaxation of the corpus cavernosum smooth muscle by activating the NO/PDE5/cGMP signaling pathway, providing a therapeutic effect on ED (Li et al., 2022). Third, osthole enhances the expression of enzymes responsible for generating H_2_S, promoting its endogenous synthesis, which aids in regulating smooth muscle relaxation and improving erectile function (Alan-Albayrak et al., 2025). Fourth, both catechin and epicatechin, as flavonoids, exhibit binding affinities for PDE5 that are comparable to those of sildenafil (Ejeje et al., 2024), which indicates that these metabolites can mitigate oxidative stress damage in endothelial and smooth muscle cells within the corpus cavernosum (Wang et al., 2024; Smith et al., 2023). Although there are no direct reports on the use of bergapten and imperatorin for treating ED, they have been proven to possess anti-inflammatory or antioxidant properties (Xu et al., 2025; Feng et al., 2025). This protective effect may also be beneficial for ED.

Furthermore, several additional active metabolites in CXC have been validated by modern pharmacological studies for their roles in treating ED. Black ants (Formicidae; *Camponotus* spp) are rich in essential nutrients, including zinc and vitamins D and E (Yang et al., 2020). Notably, black ants contain a substantial amount of zinc, ranging from 120 to 198 mg/kg (Yang et al., 2020). Research indicates that zinc can enhance testosterone synthesis and release by inhibiting oxidative stress driven by xanthine oxidase/uric acid and downregulating the inhibitory effects on the pituitary-testis axis, ultimately improving libido and erectile function in male rats (Besong et al., 2023). Vitamin D has been shown to upregulate the NO/cGMP pathway by promoting the synthesis and release of endothelium-derived NO, thereby enhancing penile erectile function (Talib et al., 2017). Additionally, vitamin E increases NO levels and superoxide dismutase activity in penile tissue by elevating intracavernous pressure/mean arterial pressure in rat models, which further improves erectile function (Helmy et al., 2012). *Lycium barbarum* polysaccharide, the primary active metabolite in Lycii Fructus (Solanaceae; *Lycium barbarum* L), has been demonstrated to enhance penile blood supply by upregulating eNOS, nNOS, and cGMP expression, while also improving corpus cavernosum function by mitigating cavernous nerve injury through increased antioxidant enzyme activity (Zhao et al., 2016; Moon et al., 2017).

### 5.4 Security evaluation

We conducted a statistical analysis of adverse events associated with the combination of CXC and PDE5Is in the treatment of ED. The overall incidence of adverse events was found to be 9.11%. The rates of specific adverse events were as follows: headache 4.15%, nasal congestion 1.61%, facial flushing 4.88%, nausea 3.60%, abdominal distension 2.11%, dyspepsia 3.48%, liver dysfunction 2.50%, rash 3.23%, dizziness 0%, and gastrointestinal bleeding 0%. The meta-analysis indicated that the adverse effects of CXC combined with PDE5Is were comparable to those of PDE5Is alone, suggesting that the addition of CXC did not significantly increase the risk of additional adverse events (RR = 0.94; 95% CI: 0.62–1.42; *P* = 0.77). Furthermore, the combination of CXC and PDE5Is did not elevate the incidence of individual adverse events, including headache (RR = 0.73; 95% CI: 0.38–1.38; *P* = 0.33), dizziness (RR = 0.33; 95% CI: 0.01–7.96; *P* = 0.50), nasal congestion (RR = 0.39; 95% CI: 0.09–1.66; *P* = 0.20), facial flushing (RR = 1.00; 95% CI: 0.26–3.87; *P* = 1.00), nausea (RR = 1.80; 95% CI: 0.39–8.26; *P* = 0.45), abdominal distension (RR = 1.33; 95% CI: 0.30–5.87; *P* = 0.70), dyspepsia (RR = 1.33; 95% CI: 0.34–5.21; *P* = 0.68), gastrointestinal bleeding (RR = 0.33; 95% CI: 0.04–3.14; *P* = 0.34), liver dysfunction (RR = 0.50; 95% CI: 0.09–2.65; *P* = 0.42), and rash (RR = 5.00; 95% CI: 0.24–102.07; *P* = 0.30). The adverse events reported during the treatment period in the included studies were generally mild and manageable. Researchers speculate that these adverse events may be related to PDE5Is, as these inhibitors can cross-react with PDE isoenzymes, leading to transient vasodilation in the heart and other organs (Schwarz et al., 2007). Currently, there is no evidence to suggest that CXC may cause additional adverse events during the treatment of ED, indicating that CXC may serve as a safe complementary therapy.

To comprehensively assess the safety of CXC, we conducted a thorough review of all RCTs reporting adverse events associated with CXC, without restricting the analysis to specific diseases. In these trials, the experimental group received either CXC alone or CXC in combination with conventional medications, while the control group received only conventional treatments. Ultimately, 41 studies were included in this analysis (Supplementary Table S1). Of these, 16 focused on the safety of CXC in treating ED, seven on prostatitis, six on polycystic ovary syndrome, five on oligoasthenospermia, four on infertility, two on premature ejaculation, and one on rheumatoid arthritis. The results of the meta-analysis indicated that CXC did not increase the incidence of adverse events such as headache, dizziness, nasal congestion, flushing, nausea, abdominal distension, indigestion, gastrointestinal bleeding, abnormal liver function, dysuria, diarrhea, palpitations, skin allergies, fatigue, dry mouth, or constipation (*P* > 0.05), as shown in Supplementary Table S2. These findings suggest that CXC is a safe therapeutic option.

It is important to note that the complication rate or adverse event rate presented in this study may not fully reflect real-world outcomes associated with the combination of CXC and PDE5Is. Firstly, the included studies in our meta-analysis were predominantly RCTs. These trials often have strict inclusion and exclusion criteria, which may lead to a selected sample that is not fully representative of the general population. For example, patients with certain comorbidities or those taking other medications may be excluded from the trials. In real-world clinical practice, patients often present with complex medical histories and may be concurrently using multiple medications, which could potentially interact with CXC and PDE5Is and increase the risk of adverse events. Secondly, the follow-up periods (4–12 weeks) in the included studies were relatively limited. Some adverse events may have a long-term latency period and may not be detected within the short follow-up durations of these trials. For instance, chronic liver or kidney function impairment may take months or even years to manifest, but the follow-up in most of our included studies was likely not long enough to capture such events. Thirdly, the reporting of adverse events in the original studies may be incomplete. There could be under-reporting of mild or self-limiting adverse events, as patients may not always disclose these symptoms to their healthcare providers, and researchers may not actively collect data on all possible adverse events. Moreover, the sample sizes of some of the included studies were relatively small. Small sample sizes may lead to insufficient statistical power to detect rare but serious adverse events. Consequently, the complication rates we calculated may underestimate the true risk of adverse events associated with the combination of CXC and PDE5Is. While our meta-analysis provides valuable information on the safety of CXC and PDE5Is combination, the complication rate presented here should be interpreted with caution. Future large-scale, long-term, and real-world studies are needed to more accurately assess the safety profile of this combination therapy.

### 5.5 Limitations and outlook

Although the results of this study contribute valuable clinical evidence for the treatment of ED with CXC, some potential limitations must be acknowledged. First, due to insufficient reporting of allocation concealment and blinding of participants and personnel in the included studies, there may be an increased risk of selection and performance bias. Future studies should enhance the description and implementation of intervention blinding to ensure the scientific validity and rigor of the study design. Second, the present study only included studies conducted in China, and the study samples were primarily from clinical centers in China, which may have led to a geographic bias. Future studies should aim to expand the geographic scope by including multiple countries or regions and incorporating patient populations from different ethnic and cultural backgrounds to enhance the generalizability of the findings. Third, although the present study evaluated the clinical effects of CXC in combination with PDE5Is, the benefits and risks of CXC alone could not be explained. Future studies should focus on the effects of CXC alone and explore its benefits and risks in ED maintenance therapy. Fourth, the TSA in the present study indicated that the sample size for adverse events did not meet expectations, suggesting that the findings from the analysis of adverse events need to be validated by additional similar studies. Future studies should strengthen the monitoring and reporting of adverse events to comprehensively assess the safety of CXC. Additionally, the long-term effects and safety of CXC need to be further explored to provide more reliable clinical guidance.

## 6 Conclusion

CXC effectively enhances erectile function and testosterone levels in patients with ED, without increasing the incidence of adverse events. These findings support the potential of CXC as a supplementary treatment for ED, with a recommended dosage of 1.26 g administered three times daily. However, due to the limited quality of the current evidence, further validation through multicenter, randomized, double-blind, controlled trials is necessary.

## Data Availability

The original contributions presented in the study are included in the article/Supplementary Material, further inquiries can be directed to the corresponding authors.
